# Sample Size and Precision in NIH Peer Review

**DOI:** 10.1371/journal.pone.0002761

**Published:** 2008-07-23

**Authors:** David Kaplan, Nicola Lacetera, Celia Kaplan

**Affiliations:** 1 Department of Pathology, Case Western Reserve University, Cleveland, Ohio, United States of America; 2 Department of Economics, Case Western Reserve University, Cleveland, Ohio, United States of America; 3 Department of Psychology, Brandeis University, Waltham, Massachusetts, United States of America; University of Exeter, United Kingdom

## Abstract

The Working Group on Peer Review of the Advisory Committee to the Director of NIH has recommended that at least 4 reviewers should be used to assess each grant application. A sample size analysis of the number of reviewers needed to evaluate grant applications reveals that a substantially larger number of evaluators are required to provide the level of precision that is currently mandated. NIH should adjust their peer review system to account for the number of reviewers needed to provide adequate precision in their evaluations.

## Introduction

On February 21, 2008 the recommendations of the Working Group on Peer Review of the Advisory Committee to the Director of the National Institutes of Health (NIH) were posted on the internet [1]. This committee made several suggestions including shortening of the application size, giving applicants unambiguous feedback about resubmission, using short pre-buttals to correct factual errors in review, and eliminating the special status of amended applications. A further recommendation of the group was to “engage more persons to review each application – “optimally 4 or more” [2].

Thus, the Advisory Committee has left the actual number of reviewers to evaluate each grant application ambiguous. No guidelines were provided to determine the number of reviewers that would be needed. Consequently, we have conducted a statistical analysis to provide guidance in arriving at appropriate numbers. Our analysis shows an inherent statistical inconsistency in the NIH peer review recommendations concerning the number of reviewers. We also demonstrate how crucial this number is and how it influences the precision of the eventual score.

## Analysis

For each grant proposal reviewers from the relevant scientific community are asked to report their evaluations within a pre-defined scale. The average grade obtained through this process is considered a valid estimate of the “true” value of the proposal.

The survey sample size is a crucial parameter in determining whether we can rely on these mean estimates. Elementary sampling techniques give us the minimum number of respondents that are needed for the evaluation procedure to deliver reliable estimates:
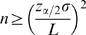
(1)In expression (1), n is the minimum required sample size or number of evaluators. Z_α/2_ is the upper percentile of the standard normal distribution. For a 95% confidence interval and an alpha (type I error, i.e. the probability of rejecting the null hypothesis when it is true) of .05, Z_α/2_ is equal to 1.96. The parameter σ represents the underlying standard deviation. Finally, L indicates the desired half-width of the interval between two consecutive evaluations or the precision of the evaluation.

There are two important implications of this equation. First, the inverse correlation between n and L indicates that more reviewers are needed to obtain a more fine-grained or precise evaluation. Moreover, this relation is exponential so that greater precision comes with an increasingly greater number of reviewers.

Second, typically the standard deviation σ of a population is not observed and needs to be estimated. Since the data necessary to estimate σ for the review of biomedical research proposals have not been collected in a statistically robust sampling system, we have relied on a model system of peer review with short movie proposals reviewed on a scale from 1 to 5 by undergraduate students [Lacetera, Kaplan, Kaplan, submitted]. We used short movie proposals in order to increase the potential sample size since all undergraduate students could be considered expert enough to grade the proposals. In this study 10 proposals were scored by an average of 48 reviewers. The average standard deviation was approximately 1.0 with a standard deviation considerably less than 0.1. Therefore, we estimate σ to be equal to 1. Obviously, a more accurate estimate of the standard deviation can eventually be obtained for each form of application requested by NIH, although it should be clear that a large number of independent evaluators is required to make any estimate of σ reliable.

Using equation (1), we can assess the effect of having 4 reviewers for each proposal. With four reviewers and a standard deviation of 1, the review would be expected to distinguish applications at the level of the unit interval:
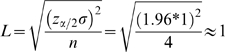
(2)Thus, four reviewers would be able to distinguish among whole integer scores.

Yet, in the evaluation of grant proposals NIH currently uses a 41-grade scale with a range of scores from 1.0 to 5.0 [3]. Moreover, these scores are averaged to yield a score with 3 significant figures instead of 2 [3]. It is this number, inappropriately expanded to 3 significant figures by averaging, that is used by NIH in their scoring decisions. Although NIH does not explain the rationale for the conversion of their scores to 3 significant figures, with 80,000 applications per year it seems likely that the NIH peer review system needs that level of precision to facilitate their making choices close to the funding line. As a consequence to the use of scores with 3 significant figures, differences as small as 0.01 are used in making funding decisions. Nevertheless, in order to obtain reliable scores with a precision level of 0.01, an unrealistically large number of reviewers would be needed:
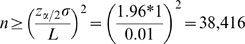
(3)Expression (3) implies that, in order for a mean score of 3.56 to be taken as reliable and therefore as identifying a better, more promising proposal than one receiving a rating of 3.57, the evaluation of almost 40 thousand referees would need to be obtained.

**Figure 1:**
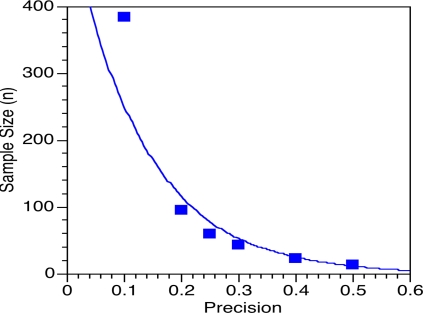
The relationship between the precision of the evaluation system (how fine-grained it is established to be) and the minimum required number of evaluators needed for reliable estimates.

In Figure 1 the exponential relationship between the number of reviewers and the precision of the ratings that would provide reliable estimates of the mean is shown. On the x-axis, smaller numbers indicate higher precision. Even for a precision level of 0.1, as many as 384 reviewers would be required.

The disconnect between the needed precision in order to allocate funds in a fair way and the number of reviewers required for this level of precision demonstrates a major inconsistency that underlies NIH peer review. With only four reviewers used for the evaluation of applications, an allocation system that requires a precision level in the range of 0.01 to 0.1 is not statistically meaningful and consequently not reliable. Moreover, the 4 reviewers NIH proposes are not independent which degrades the precision that could be obtained otherwise.

Consequently, NIH faces a major challenge. On the one hand, a fine-grained evaluation is mandated by their review process. On the other hand, for such criterion to be consistent and meaningful, an unrealistically high number of evaluators, independent of each other, need to be involved for each and every proposal.

Further insights can be derived from the analysis of expression (1). The value of σ is a measure of the underlying variability in the ratings. The minimum number of reviewers for any given degree of ratings precision decreases with decreasing standard deviations. The standard deviation across ratings is also an indicator of the degree of agreement among different reviewers. If the standard deviation is small, for instance equal to 0.01 instead of our previous working estimate of 1.0, there is essentially consensus among the referees. If σ = 0.01, then the following relation holds:
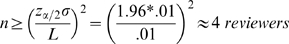
(4)Therefore, 4 independent evaluators can provide statistical legitimacy only under the circumstance of all evaluators giving essentially the same evaluation. For proposals that are expected to be more controversial, as potentially transformative ideas have been proposed to be^5^, a small number of evaluators would lead to unreliable mean estimates.

**Figure 2:**
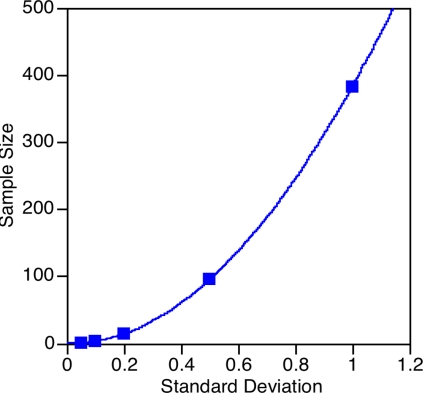
The relationship between the standard deviation of the scores and the minimum required number of evaluators needed for a precision of 0.1, which is the level of precision currently obtained in the NIH peer review system.

Our estimate of σ is not based on an analysis of biomedical research experts judging research projects close to their area of specialty. Scoring standard deviations for large numbers of experts obtained in a statistically acceptable sampling system have not been collected. Instead, as described above, we have used a model system that has allowed us to readily collect opinion data about proposals with undefined potential. Although we believe our estimate is reasonable, it is informative to visualize how the sample size estimate varies with different values of standard deviation for a level of precision of 0.1 (Figure 2). It is evident that small sample sizes are able to provide levels of precision only when the standard deviation is exceptionally small. We used a level of precision of 0.1 because the NIH peer review system mandates scoring at this precision level. For greater levels of precision, as suggested by the conversion from 2 significant figures to 3, the increase in sample size is steeper with increasing standard deviation.

**Figure 3:**
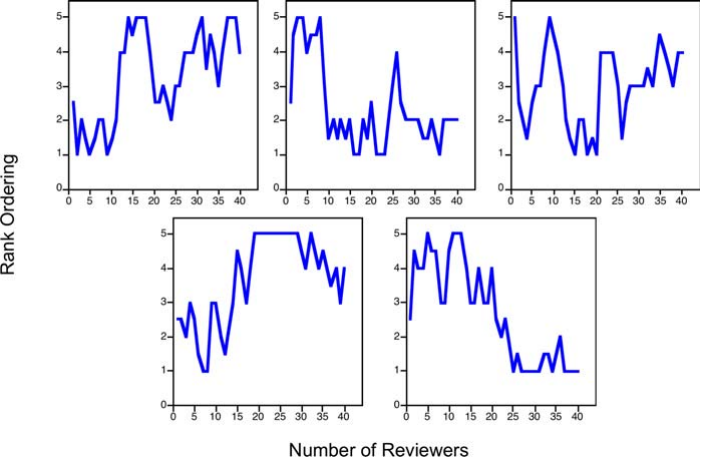
Five individual movie proposals were evaluated by 40 reviewers and the rank ordering of the proposals was assessed as reviewers were randomly included in the analysis. The 5 proposals were closely spaced with mean scores of 3.46 to 3.64. Proposals that had the same score were given an averaged rank; the figures changed little by assigning proposals with the same score the highest *ranking*.

The importance of scoring accuracy ultimately relates to the rank ordering of proposals. In our model system there were 5 movie proposals with mean scores ranging from 3.46 to 3.64. We have analyzed how the rank ordering of these 5 proposals varied as reviewers were randomly included in the analysis from 1 to 40 reviewers (Figure 3). What is most striking in these graphs is the extreme variability in the rank ordering with low numbers of reviewers. For instance, the upper-left and lower-left panels of Figure 3 show proposals that had relatively good rankings with less than 10 reviewers but that ended with relatively poor rankings with over 30 reviewers. Conversely, the lower-right panel shows a proposal that began with poor rankings but settled at with the best ranking after 25 reviewers. Even the addition of 1 reviewer can markedly change the rank ordering of the proposals and consequently the funding decision. This effect is especially apparent when there are few reviewers. The number of reviewers has profound implications in terms of the actual funding decisions that are eventually made.

## Discussion

It is clear from our analysis that NIH needs to adjust their peer review system to account for low precision evaluations. Additionally, it would be valuable to determine the standard deviations of scores given by independent reviewers. This information could be used to obtain more appropriate estimates of σ and consequently would be invaluable in designing and implementing a statistically rational system of social choice for NIH.

Our data demonstrate that funding decisions will vary widely with the number of reviewers in considering proposals that are closely scored. Making choices between applications that vary by less than 1 will require larger numbers of reviewers than NIH has been contemplating. Recognition of the statistical inconsistencies of NIH peer review will allow for the implementation of new policies that take into consideration the accepted relationship between the number of reviewers, the precision of scoring needed, and the standard deviation of the scores given.

The Working Group also recommended shortening the length of the application although no specific suggestions were included^2^. Obviously, the length of the application impacts the number of reviewers that could possibly be used for scoring. More reviewers can be used for shorter applications.

It is commonly accepted that NIH will not fund clinical trials that do not include a cogent sample size determination. It is ironic that NIH insists on this analysis for clinical studies but has not recognized its value in evaluating its own system of peer review. We posit that this analysis should be considered in the revisions of NIH scientific review.

The NIH peer review structure has not been based in rigorous applications of statistical principles involving sampling [4]. It is this deficiency that explains the statistical weakness and inconsistency of NIH peer review. Although NIH has made an excellent effort to remedy some of the most egregious problems inherent to their peer review system, the Working Group has neither fully realized nor addressed the statistical problems that have beset the NIH peer review system.
